# Extra Supplement—The Nursing Mirror

**Published:** 1890-05-03

**Authors:** 


Being the Extra Nursing Supplement of "The Hospital" Newspaper.

All Contributions for this Supplement should be addressed to the Editor, The Hospital, 140, Strand, London, W.C., and should have the word "Nursing" plainly written in left-hand top corner of the envelope.

### En Passant.

BRITISH NURSES' ASSOCIATION.—We have been requested to make the corrections embodied in the following letter from Sir Andrew Clark, F.R.S., President of the Royal College of Physicians: "My attention having been called to several reports of a meeting of the British Nurses' Association said to have been presided over by "Dr. Andrew Clark," and being the only person in the Medical Directory entitled to that designation, I request permission to say, in your next issue, that I am not connected with that institution, and that I have never presided over any of its meetings."

UNIFORMS FOR JUNE.—Our correspondents seem to have fallen into the mistake of recommending indoor uniforms: the presentations will take place in the grounds of Marlborough House, and the nurses will all be in out-door uniform. Any neat cloak and bonnet in a quiet colour will do; veils look nice, so do white strings to the bonnets. Those nurses who live in out-of-the-way districts may care to know that Wallis, of Holborn Circus, and Shoolbreds, both make a specialité of items of nursing costumes. Cloaks, ready made, cost about 15s. 6d.

NURSES' MISSIONARY ASSOCIATION.—A society of nurses has lately been started under the above name, with the object of providing medical and nursing comforts for the sick, not in England, but in less favoured countries abroad. The medical missionary needs supplies; his skill in treating bodily diseases, and even in easing the tortured mind, is decreased without proper appliances and comforts for his patients. Hence nurses, who know so well the trials of sickness, have determined to do their best for foreign missions, by making nursing appliances their special charity. The hon. sec. of the association is Miss D. M. Image, 53, Gloucester Street, S.W.

STAFFORDSHIRE INSTITUTION.—The eighteenth annual report of this institution tells of plenty of good work accomplished. The present staff consists of 68 nurses engaged in private nursing, 12 nurses engaged in district nursing, and 12 probationers in training. During the year 500 cases have been attended, and many have had to be refused, no nurses being available at the time they were needed. 28 cases were nursed on reduced terms for a period of 160 weeks, and 32 gratuitously for 84 weeks. In spite of all this gratuitous work and the fact that the nurses are well paid and made very comfortable, a handsome balance is carried forward to next year. The home has recently been enlarged, but the proposal to build a home for paying patients has been abandoned for the present. As usual, the hearty thanks of all are given to the Lady Superintendent.

RICHMOND INSTITUTION.—Encouraged by the success of their institution at Blackheath, Mrs. Smith and Miss Duncan have opened a similar home for private nurses at Richmond, Surrey. Miss James, as Sister-in-Charge, and five nurses have taken up their quarters at the new home, and are already hard at work.

AMERICAN PAPERS.—We have constantly given a good word to that excellent American paper for nurses, *The Nightingale*; but we have no similar commendation for *The Trained Nurse*. This latter paper gets itself up in handsome garb, but is nothing more than a whited sepulchre! Its contents are all dead bones, or at least old articles filched wholesale and without acknowledgment from the pages of The Hospital, *Nursing Notes*, and other papers. Here is the whole of Dr. Percy Lewis's chapter on "Chest Diseases," here is the whole of Mrs. Hewer's paper on "Bed Sores." We beg to remind our American friends that though there is no copyright between the two countries, there is generally supposed to be a certain amount of honesty among all human beings, and that for our part we should scorn to descend to such journalistic methods.

SHORT ITEMS.—Miss Wylde has decreased the house expenditure, and resuscitated the nursing home at Chichester Infirmary.—The Society of Apothecaries of London offers two prizes to young women under twenty years of age, who are students of botany, for proficiency in that science. The examinations, written and oral, will take place on Wednesday, 25th, and on Saturday June 28th.—The Duchess of Westminster will distribute the prizes for the Home Nursing Class at Grosvenor House on May 12th.—The *Illustrated Military and Naval Magazine* for May contains an article on "Her Majesty's Nursing Sisters."—Our many readers will be glad to hear that Miss K. M. Heanley is progressing towards health.—Dr. Downs sent a class of thirty-five ambulance pupils up for examination at Eastbourne and only two failed.—Nurse Tomson of Guildford Hospital has designed a child's sleeping suit, and sent a doll dressed in a specimen suit to the bazaar for the new Hospital for Women.

A SUGGESTION FOR THE CAPE GOVERNMENT.—We have received a letter from "Franciscus," asking if Government is going to employ Catholic nurses at the new leper hospital on Robben Island. She says: "Several of us Catholic nurses enter convents, and I think if some of us trained from now in all details most useful in leper nursing, and gave ourselves definitely to the work, the Government might feel disposed to accept us. I offered myself for this work some years ago, and our Cardinal's permission was asked, but circumstances prevented the carrying out of the original plan. Anyhow, I hope to enter some convent soon, and my idea is that those of us who wish to go, if accepted, should go as nuns, as, though you probably have no sympathy with the Roman Catholic religious aspect of it, you will see that there are advantages in a community of women, wanting nothing for themselves but bare necessaries, dedicated to their work for life, and having perfect order and discipline in themselves. Of course, this is merely a preliminary step; the second step would be to get some Catholic order to receive and train us." "Franciscus" is an old correspondent of ours, and thoroughly trained in general and district nursing. Her idea is certainly worthy of consideration, and we shall take care it is made known in the proper quarters.

### Nursing Lectures.

By Percy Lewis, M.D.

### Lecture X.—THE SKIN.

The skin consists of two layers, the true skin containing the blood-vessels, nerves, glands, and hair follicles, and the cuticle or epidermis covering it. It is the latter which is shed in scarlet fever in large flakes. Normally the outer layers are always being shed or rubbed off by the clothes as fine scales, and being replaced by others. The glands are the sweat glands secreting the perspiration, and the sebaceous secreting an oily substance, which gives to the skin and hairs their natural gloss. The nerves end for the most part in microscopic conical elevations of the true skin called papillæ, and are the organs of touch. The skin has three functions: it is an organ of sense, of excretion, and a regulator of the heat of the body. In the last lecture we found that the skin and the kidneys were nearly related in function, and that when the secretion of one was increased that of the other was diminished. Perspiration is always more or less going on, generally so slowly that it evaporates as quickly as produced, and is spoken of as "insensible" perspiration; when, however, it is secreted more quickly than it can be evaporated, it is called "sensible" perspiration.

The skin is subject to many diseases with which, as far as nursing goes, you will have little to do. A rash must be reported whenever it occurs, especially if a patient admitted for something else is found to have a rash on being undressed. Soap should never be used in skin diseases without permission, as it causes irritation.

Applications used in the treatment of skin diseases may be moist, dry, or greasy. A moist application is meant to be moist and kept moist, not allowed to be dry and moist alternately, for this will do more harm than good. Lead lotion and water dressing are used as moist applications, either being allowed to drip continually on lint covering the surface, or else the lint wetted with them is covered with oilsilk. Covered with oilsilk they form warm applications; uncovered, cold ones.

When told to remove scabs from the skin, in default of orders to the contrary, proceed thus: Pour oil on them, and cover with lint soaked in oil for two or three hours; then apply a linseed poultice. On removing this at the end of three or four hours, you will probably find that the scabs can be lifted off without discomfort; still, avoid irritating the skin by picking and pulling at firmly-attached ones. Ointments should be spread on lint; not thickly, so as to leave cakes of ointment on the skin when the lint is removed, but finely and evenly. Over the lint a thin layer of cotton wool should be placed, and the part evenly bandaged, so as to bring the ointment in close contact with the skin. Remember that chrysophanic acid will stain any linen it may come in contact with a bright yellow colour, for which reason it is necessary to keep separate sheets for patients using this ointment. Of course, the better applied are the bandages, the less likely is the ointment to come in contact with the sheets. In diseases of the ear or nose, oil and ointment are applied on the outside by plugs of lint.

The skin requires extra attention in cases such as phthisis, dropsy, or paralysis, where patients have to spend weeks in bed, for, as you know, they are very liable to be attacked with bed-sores. Nurses should know how to prevent bed-sores, because it is always considered a sign of bad nursing when one occurs. Pressure must be relieved from all the more sensitive and prominent parts, as sacrum, elbows, knees, ankles, by circular pads; these parts should be washed night and morning with soap and water, well dried, and afterwards dusted with starch or oxide of zinc powder. All moisture, crumbs, and wrinkles in the bed must be avoided; the patient's position must be frequently changed, and water cushions used as much as possible.

If, however, the skin gets red in spite of every care, it must be well washed two or three times a day with spirit, allowing this to dry on. Should the skin break, paint collodion over the broken part, but continue to use the spirit over the unbroken part. In case of the sore getting worse, the responsibility of treating it devolves on the medical man, to whom you will, of course, have announced its first appearance. Parts which cannot possibly be kept dry should be well rubbed over with oil or lanoline.

Many kinds of baths are used in the treatment of skin disease; before mentioning them, however, I will give you the rules which should guide you in giving a bath: (1) Everything should be ready for giving the bath before disturbing the patient; (2) the temperature ordered should be maintained by constantly adding more hot water at the side and testing with a bath thermometer; (3) never leave a heart case or a convalescing case in a bath by themselves, for both are liable to fatal syncope; (4) the time should not exceed ten minutes, unless specially ordered; (5) avoid chill after by quickly drying and returning to bed.

The temperature of a cold bath should be about 70 deg., a tepid bath 85 deg. to 92 deg., a warm bath 92 deg. to 98 deg., and a hot bath 98 deg. to 110 deg. In hip baths the water will cool much more quickly, consequently they require the hot water adding oftener. It is desirable, in order to prevent the patient's body becoming cold, that a blanket should be thrown over the shoulders. A bran bath is made by boiling 4 lbs. of bran with a gallcn of water, straining, and adding the strained infusion to an ordinary warm bath. A salt bath by adding 1 lb. of common salt to every four gallons of water; a sulphur bath by adding 2—6 oz. of sulphide of calcium; an alkaline, of 2—4 oz. of carbonate of sodium or potash; and a mustard, by adding a handful or two of mustard to an ordinary bath.

To make a vapour bath, the patient may be seated in a chair and surrounded with blankets from his neck to the ground. Under the chair is placed a shallow basin or dish containing boiling water to the depth of two or three inches, into which red-hot bricks are dropped. A hot-air bath may be given in the same way, substituting a spirit lamp with a large flame for the basin and hot bricks; both vapour and hot air baths are, however, more often given to the patient in bed, with a special apparatus which I need not now describe. A hot pack consists in wrapping the patient up in a hot, wet blanket or sheet, and covering him with dry blankets, which are tucked in all round him. A cold pack is similar, except that the wet blanket is cold. In both cases a mackintosh covered with a dry blanket is placed between the patient and the mattress, and all body linen is removed before the pack is given. As it is important to avoid chill after the pack is removed, the patient should be kept for half an hour or so wrapt up in a dry blanket, then rubbed dry with a warm towel and covered with the ordinary bedclothes.

For any other kind of bath than those mentioned which may be ordered, you will receive special instruction from the medical man in charge of the case.

### Death in our Ranks.

Miss S. A. Cross.—Died on Monday, April 15th, at the Derby General Infirmary, of typhoid fever, Miss S. A. Cross, daughter of Mr. R. Cross, J.P., of Oxford. Miss Cross was trained at the London Hospital in 1885 to 1887, and afterwards for some months took up district work. In May, 1889, was appointed night superintendent at the Derby Infirmary, which post she held for about four months. Since then, until her illness, she has been in charge of the Alexandra wards; her death has caused a blank at the infirmary, where she was beloved and esteemed by all. She was interred at Oxford on Saturday, the 19th inst.

Miss M. V. Edwardes.—On the 20th inst., of pneumonia, died Mary V. Edwardes, who had been a probationer at the London Hospital since October, 1888. Miss Edwardes was only 25 years of age, and is deeply regretted.

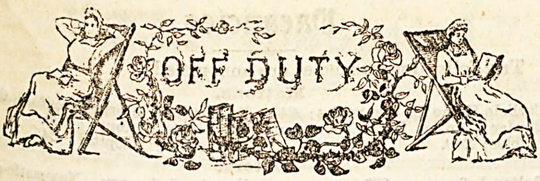

### The Passing of the Shadow.

III.

It must have been about five years since the events recorded above, that I went with these friends on a picnic] down the river near the chateau. Time had brought but little change to the quiet family, over which still hung the mystery of the undiscovered crime; the proud Sophie was Comtesse de Moreau, but Celimène had remained with the fragile Annette, whom she loved too well to leave. We were a very merry party, about twenty in all, but only the younger ones among us went by boat, our host and hostess and their contemporaries having selected the shorter overland route. I happened to be sitting near Annette, who had grown much paler and quieter, I thought, since I saw her last.

"How beautiful the country looks with the autumn sun shining upon it, Mademoiselle," said I, with keen appreciation of the gliding movement, the warmth, the scenery, and the bright faces.

"O do not talk about the sunshine," said Annette, "I cannot bear it."

"It always strikes me," I began again, "that there is something eminently French about the scenery in this province. Those little green trees and houses on the hill look so bran-new and clear-cut that they might well have been just taken out of a Noah's Ark, and put up there for us to look at! Even your rabbits have the defined outline of the reflection of a child's fingers by candlelight."

"For Heaven's sake do not talk about reflections," said Annette, and I saw that this time I had really pained her.

"Has it ever occurred to you what a funny crooked old world it is, Mademoiselle," said I, with a desperate effort. I confess that I hate to carry on conversation in shallows like this—I like a good broad stream of talk to roll on without a pause; and Annette was not an inspiring companion that day. "And how many funny crooked old people there are in it, and that all its pathos and picturesqueness comes from the fact that it is tumbledown and crooked?"

No answer. This was rather a severe tax on the imagination. "Look at that rugged old boatman, for instance. His dark, knotted hair has grown all over his face, and his skin is baked the colour of a brick. And just for this reason, because he is wild and unkempt, you cannot fail to look upon him without pleasure."

"Please, Monsieur, do not speak of him; there is something about him that repels me, and I feel in my own mind that I am doing an injustice to the poor man. Do not think me rude, but I am not well to-day."

I took the opportunity, later on, after we had landed, of telling Celimène of her sister's indisposition. "You have probably touched on some sore point with her," replied she. "Annette has never been the same since the night of my brother's murder." Then, in the course of conversation, she told me all the details that I have written above.

"You believe the man who crept through your sister's room to have been the murderer?"

"We do," replied she.

"And you identify him with the adventurer Count?"

"Most certainly."

"What object can he have had?"

"Either revenge, because my brother betrayed who he was, and so robbed him of a prize, or for money, as none was found on my brother's person. Probably for both reasons."

"And he has never been discovered?"

"No," she replied.

"Nor ever will be," said I.

"Who can tell," said Celimène, "I do not give up hope."

How well I remember the scene! In front of us was a pretty backwater of the river, with a ford across it, leading to a tiny island, overgrown with underwood, where some of the guests had been rambling. Behind were the soft grassy hills, over which the sun was brooding, as if loth to sink. In the foreground young men and ladies were reclining on the sandy soil, through which stray blades of grass made their way, gave the effect in the distance of white silk shot with green. Annette was sitting somewhat apart, looking at the ground—Sophie was telling the boatman I had noticed to fetch something from the island—her parasol, I think, which she had left there. Celimène was amusing a group of friends. Presently I walked to where Annette was sitting. As I approached I saw her give a violent start, and heard her utter an exclamation. The level sun was casting long shadows upon the soil, and as I stood in the shade, I saw a great black shadow pass in front of her, and move slowly onwards. I turned to see what body made it. It was only the boatman as he crept from stone to stone in the ford; but I confess that the movement of the flat mass created in me a strange repugnance. It was different with Annette. She recognised the movement. It was one not easily mistaken. With almost supernatural effort she rose, turned round, and fell in a dead faint at my feet.

I do not know in how far it increased her horror to feel that for five years she had been in constant intercourse with her brother's murderer; I do not know how the adventurer Count succeeded in entering the service of the family he had so fatally injured—it was doubtless the cleverest thing he could have done to remain on the spot, where he would be least suspected—but I know this, that he was arrested, proof was found of his guilt, and he was condemned.

M. E. Wheeler.

### Everybody's Opinion.

THE JUNIUS S. MORGAN MEMORIAL.

Nurse Wilson writes: I have already acted upon your suggestion that we should raise a Memorial Fund, and have quite a nice little sum gathered towards my £5. I think it is a splendid idea, and surely when so much has been done for us we ought to feel an indescribable pleasure in rendering a little assistance to our more unfortunate sisters. I feel sure all our sisters of the first thousand will gladly "put their shoulder to the wheel," and gather together perhaps even more than the £5,000 desired.

"One of the First Thousand" writes: The suggestion in this week's Hospital for the Junius Morgan Memorial is so much better than my own that I trust you will allow me to withdraw my last letter. I doubt whether the first thousand —or most of them—can collect £5 a-piece between this and the end of June. The friends of professional nurses, as distinct from the lady players, are seldom wealthy, and it is not every rich patient one would care or dare ask for a subscription. Collecting cards would be useful. They have a business-like appearance, and might be worded to explain the object of the benevolent fund, and save us the awkwardness of seeming to beg for ourselves.

[Collecting Books are being prepared. Application may be made to the Secretary, National Pension Fund for Nurses, 8, King Street, Cheapside, E.C. One or more nurses may combine to make one purse. Each nurse can at least do something in this way.]

CERTIFICATES, NOT POLICIES.

"F. T." writes: It is not the case that the first thousand will all receive their policies from the hand of the Princess, or, at any rate, signed by her, for I was one of the earliest (No. 195), and I received mine the other day. Will you publish this, to see if any other nurse has already received hers—the one they call the permanent one, on parchment, and signed by three people, in which the Princess's name is conspicuous by its absence?

["F. T." has, of course, made a mistake, which we hope is not shared by our other readers. The Princess will give special certificates, artistically designed, not the ordinary policies. Every one of the first thousand will possess the certificate over and above the policy.]

### Presentations.

Devonshire Square Institution.—Mrs. Keeley, who for many years so ably filled the post of lady superintendent of the Nursing Sisters' Institution, Devonshire Square, has been presented on her resignation with a purse containing fourteen guineas by the matron and sisters of the institution.—The Marchioness of Tavistock has succeeded Lady Victoria Buxton as president of the committee of this institution.

Grimsby District Hospital.—In connection with the changes which are now taking place in the Grimsby and District Hospital a pleasing presentation was made to the late matron, Miss Mountford, on Monday morning, by members of the medical staff and managing committee. The presentation was made by Dr. Moody as senior surgeon, and consisted of an address and a purse containing a handsome sum of money. The departure of Miss Mountford is regretted by all who met her in the discharge of her duties at the hospital as well as by a large circle of private friends.

Royal Sea-Bathing Infirmary.—There was an interesting gathering at this Institution on Thursday last, when all the staff, sisters, and nurses met to congratulate Sister Alexandra on her completion of 25 years' nursing work in the Hospital. The Directors, to mark their appreciation of the valuable services rendered by Sister Alexandra, presented her with a cheque for £25, which was handed to her by the Matron. The many friends of Sister Alexandra will be glad to hear how much her devotion to her work is appreciated.

Redditch Ambulance Association.—After the presentation of medallions and certificates to the successful pupils at the winter session of the Redditch St. John Ambulance Association, an interesting ceremony took place. The Chairman said he had much pleasure in announcing that Mrs. Bartleet and Mrs. Allcock had been deputed by the members of the class to make a present to Dr. and Mrs. Page as a token of their appreciation of their valuable services during the last three years. The presentation, which consisted of a richly-chased gold bracelet set with pearls for Mrs. Page and a handsomely-engraved silver salver for Dr. Page, was then made by Mrs. T. Bartleet, who said: Dr. and Mrs. Page, Mrs. Allcock and I have been deputed by the ladies who have attended your lectures to express their grateful appreciation of the trouble you have taken on their behalf. We are all deeply sensible of the obligation we are under to you, for the pains you have taken over our instruction, and for the time which you have ungrudgingly devoted to us. We also desire to thank Mrs. Page very heartily for her kindness in acting as our coach. We have much pleasure in begging you and Mrs. Page to accept these small tokens of our regard, which we offer with our very best wishes for the health and happiness of you both.

Staffordshire Institution.—On February 8th the Bishop of Shrewsbury, in the name of the nurses of the Staffordshire Institution, Stoke-on-Trent, presented Miss Shirley, the Lady Superintendent, with a very handsome gold watch, with her monogram and the following inscription, "Presented to Miss Shirley by the nurses of the Staffordshire Institution, as a token of love and esteem."

### Notes and Queries.

### Queries.

(6) *Ignoramus.*—What should a trained nurse charge per week for the care and maintenance of a child of 15 months, in order to wean it?

(7) *Home Wanted.*—Near Bath for an epileptic patient who could pay about 6s. a-week. *S. E. M.*

### Answers.

*Nurse Connell.*—Outdoor.

*Miss Macpherson.*—The address is printed in pamphlet form. Bishop Westcott's book is published by Rivingtons, of Waterloo Place.

*A. F.*—You must wait a little.

*Helen.*—Try Leeds Nurses' Institution, or Sheffield Union, or the Workhouse Infirmary Nursing Association.

### Vacancies.

The following vacancies are announced:

### Matrons.

Carlisle Home for Incurables; £40.

Border Counties Home for Incurables; £40.

Folkestone Victoria Hospital; £50.

St. Mary's, Tenbury; £30.

Tewkesbury Hospital; £45.

### Sisters.

Bolton Infirmary; £25.

Cardiff Infirmary; £28.

Pendlebury Hospital; £30.

Royal Infirmary, Newcastle-on-Tyne; £30.

### Nurses.

Arbroath Poorhouse; £25.

Bangor Nursing Institute; £25.

Border Counties Home for Incurables, Carlisle; £25.

Birkenhead Boro' Hospital; £25.

Blackheath Institute.

Brixton and South London Institution for Nurses.

Brixton Institute.

Carlisle Fever Hospital; £20.

Carmarthen Infirmary; £23.

Chelsea Infirmary; £18.

Church Deaconess' House, Chester; £24.

Clayton Hospital; £25.

Croydon Hospital; £25.

Darlington Fever Hospital; £26.

Dorchester Hospital (night); £22.

General Nursing Institute.

Glasgow Sick Poor Association.

Hanwell Ophthalmic School; £26.

Kilmarnock Infirmary; £14.

King's Lynn Hospital; £20.

Longton College Hospital; £20.

Leeds Institution.

Mr. Wilson's Institution.

Newcastle Infirmary; £20.

Newlands, Ryde.

Redhill Hospital (night); £20.

Royal Portsmouth Hospital; £28.

St. Helen's Cottage Hospital.

St. Helena Home.

St. Mark's Hospital, City Road, E.C.; £25.

St. Leonards Institution.

Stratford-on-Avon Hospital; £20.

St. Saviour's Infirmary; £20.

Watford Union; £25.

Weston-super-Mare Hospital (night); £25.

Workhouse Infirmary Nursing Association.

### District Nurses.

Bolton; £30.

Camborne District Nurses' Fund.

Chailey, Lewes; £30.

Cambridge Nurses' Home.

Edinburgh Queen Victoria Institute; £30.

East London Nursing Society.

Gloucester Nursing Society; £25.

Jersey Institute; £25.

Tewkesbury (midwife); £52.

Worcester City and County Institution for Nurses.

### Probationers.

Highgate Convalescent Home.

Hospital for Children, Gateshead-on-Tyne.

H.M. Hospital, Stepney.

Poplar Accident Hospital.

Wallasey Cottage Hospital.

### Appointment.

Warwick.—Miss Alice Gazey, who trained at the London Hospital, and has been working at the Jersey Institution and the Bedfordshire Institution, has been appointed first district nurse at Warwick. Nurse Gazey will have to work under two lady superintendents and 16 visiting ladies!

### Amusements and Relaxation.

### N.B.—New Word Competition Commenced April 5th, 1890, ends June 28th, 1890.

Three prizes of 15s., 10s., 5s., will be given for the largest number of words derived from the words set for dissection.

Proper names, abbreviations, foreign words, words of less than four letters, and repetitions are barred; plurals, and past and present participles of verbs, are allowed. Nuttall's *Standard* dictionary only to be used.

N.B.—Word dissections must be sent in WEEKLY to 140, Strand, W.C., arranged alphabetically, with correct total affixed.

The word for dissection for this, the FIFTH week of the quarter, being

"ACADEMY."

| Names. | Ap. 24th. | Totals. | Names. | Ap. 24th. | Totals. |
|---|---|---|---|---|---|
| Adeline | 44 | 85 | Gladys | 68 | 116 |
| Patience | 68 | 118 | Agamemnon | 65 | — |
| Lightowlers | 63 | 111 | G. E. J. C. | — | — |
| Canary | 46 | 86 | S. E. A. | — | 24 |
| M. E. S. | — | 37 | Jenny Wren | 64 | 114 |
| Ambition | — | 36 | Multum in parvo | — | 32 |
| M. W. | 64 | 114 | Ecila | — | 39 |
| Esperance | 64 | 114 | Weta | 52 | 78 |
| Judy | — | 37 | Henri | 51 | 95 |
| Reldas | 66 | 115 | Holland | 65 | 115 |
| Merenda | — | 29 | T. J. | 45 | 78 |
| Lucy Locket | — | — | Nurse Isabel | — | 17 |
| Camellia | — | 30 | Nurse Faith | — | 9 |
| Qu'appelle | 61 | 111 | E. S. Z. | — | 10 |
| Tinie | 69 | 119 | G. P. | — | 10 |
| Nurse Mildred | 54 | 87 | Stumps | — | — |
| Coralie | 68 | 116 | Bolton | — | 8 |

### Notice to Correspondents.

Stumps.—Total for first week, 23.

N.B.—Each paper must be signed by the author with his or her real name and address. A *nom de plume* may be added if the writer does not desire to be referred to by us by his real name. In the case of all prize-winners, however, the real name and address will be published.

Competitors can enter for *all* quarterly competitions, but no competitor may take more than *one first prize* during the year.

